# Is four-dimensional CT angiography as effective as digital subtraction angiography in the detection of the underlying causes of intracerebral haemorrhage: a systematic review

**DOI:** 10.1007/s00234-019-02349-z

**Published:** 2020-01-04

**Authors:** C. E. Denby, K. Chatterjee, R. Pullicino, S. Lane, M. R. Radon, K. V. Das

**Affiliations:** 1 Neuroradiology Department, The Walton Centre NHS Foundation Trust, Lower Lane, Fazakerley, Liverpool, L9 7LJ UK; 2 Royal Liverpool University Hospital, Liverpool University Hospitals NHS Foundation Trust, Prescot Street, Liverpool, L7 8XP UK; 3 Countess of Chester Hospital NHS Foundation Trust, Liverpool Road, Chester, CH2 1UL UK; 4 Department of Biostatistics, University of Liverpool, Liverpool, L69 3GL UK

**Keywords:** Four-dimensional CTA (4D-CTA), Digital subtraction angiography (DSA), Intracerebral haemorrhage (ICH), Haemorrhagic stroke, Vascular abnormalities, Systematic review

## Abstract

**Purpose:**

To determine whether the sensitivity and specificity of four-dimensional CTA (4D-CTA) are equivalent to digital subtraction angiography (DSA) in the detection of underlying vascular abnormalities in patients with intracerebral haemorrhage (ICH).

**Methods:**

A systematic review of studies comparing 4D-CTA with DSA in the detection of the underlying structural causes of ICH was performed on the literature published between 1998 and 2019.

**Results:**

We identified a total of 237 articles from PubMed, SCOPUS and Web of Science using the following Medical Subject Headings (MeSH) terms: primary intracerebral haemorrhage, 4D-CTA, DSA, cerebral haemorrhage, angiography, digital subtraction, arteriovenous malformations, 4D, CTA, dynamic-CTA and time-resolved CTA. Following the removal of duplicate publications and articles failing to meet our inclusion criteria, there were four articles potentially viable for analysis. Therefore, there were not sufficient studies to provide a statistically meaningful meta-analysis.

**Conclusion:**

The review of current literature has demonstrated that there are few published studies comparing 4D-CTA with DSA in spontaneous ICH, with only four suitable studies identified for potential analysis. However, due to the restricted number of patients and high sensitivity and specificity of 3 studies (100%), performing a meta-analysis was not meaningful. Qualitative analysis of the data concluded that 4D-CTA has the diagnostic potential to replace invasive DSA in certain cases with vascular abnormalities. However, further research studies directly comparing 4D-CTA with DSA using larger prospective patient cohorts are required to strengthen the evidence base.

## Introduction

Spontaneous primary intracerebral haemorrhage (ICH) accounts for 8–30% of strokes worldwide and is associated with high mortality (with a rate of approximately 50% within the first month) and morbidity (dependency rate of 80% presenting 6 months from onset) [1–3]. Haematoma expansion following a spontaneous ICH is one of the leading reasons behind this high mortality and morbidity, with the risk of expansion potentially persisting for several hours or even longer after the index event resulting in further expansion of existing haematoma, producing neurological deterioration [3].

Although the most common cause of primary ICH is hypertension [4], ICH secondary to treatable underlying cerebrovascular pathologies such as aneurysms, arteriovenous malformations (AVM), dural arteriovenous fistulae (DAVFs) and arterial and venous system occlusive disease are not uncommon [5], with 15–20% of patients having an underlying macrovascular cause [6]. The underlying pathologies causing ICH are complex and require both accurate and timely diagnostic workup prior to neuroradiological intervention (e.g. embolisation) to facilitate the best clinical outcome.

Historically, digital subtraction angiography (DSA) has been the gold standard for locating vascular abnormalities and aneurysms, retaining a significant role in planning for future intervention. Despite DSA being the current gold standard, there are risks associated with this procedure due to invasive arterial access and transient or permanent neurological complications. Computed Tomography Angiography (CTA) has the capability to identify the secondary causes of ICH, such as vascular malformations and arteriovenous fistulas, and is minimally invasive compared with DSA [5]. Additionally, CTA can identify patients at high risk of haematoma expansion, with a marker known as the “spot sign” [7]. The spot sign predicts the growth of the haematoma and the potential for a re-bleed, which can determine the risk of neurological deterioration, clinical outcome and mortality [8]. There have been significant advances and improvements in the CTA procedure in recent years, including the introduction of three-dimensional (3D-CTA) and latterly four-dimensional CTA (4D-CTA), which have both supplemented and to some extent replaced DSA in the diagnosis of aneurysms in certain cases, such as those with middle cerebral artery (MCA) aneurysms [9–11]. 4D-CTA, also known as time-resolved or dynamic-CTA, is now more readily available and builds upon the existing 3D-CTA stereoscopic imaging and has the time-resolution component of DSA for evaluating cerebral haemodynamics in a variety of cerebrovascular conditions [5]. In addition to 4D-CTA being non-invasive, further benefits include a reduction in the complications associated with endovascular procedures such as DSA and shorter acquisition times. This renders the technique an ideal modality for emergency cases, particularly in patients presenting with a large haematoma. 4D-CTA also enables the detection of parenchymal abnormalities such as hydrocephalus and brain haematomas that are difficult to visualise using DSA [12].

However, 4D-CTA is multi-phasic and therefore has previously been associated with higher radiation doses than the standard single-phase CTA examinations, although recent advances in this technique have reduced procedural radiation dose. A study by Radon et al. [13] demonstrated that the radiation dose can be potentially reduced by the more precise matching of exposure duration to cerebral circulation time [13]. The effective radiation dose in this study was reduced from 6.9 to 3.4 mSv. Other studies recorded radiation doses of less or equal to 5.3 mSv [12, 14, 15], which is a significant dose reduction.

Despite 4D-CTA demonstrating great potential in the assessment of AVM and other vascular abnormalities due to time resolution, there are still limitations. This review has been performed with the aim of gathering together and evaluating the literature with aims of performing a meta-analysis in order to determine whether 4D-CTA is as sensitive and specific as DSA in the detection of underlying structural abnormalities that are responsible for haemorrhagic stroke in patients with ICH.

## Materials and methods

### Design

A systematic review was conducted according to the guidelines developed by the Cochrane Database of Systematic Reviews. The research question was developed using the PICO framework [16]. The ICH patient group (> 18 years of age) constituted the Population. The Intervention is 4D-CTA, with the Comparison technique being DSA. Outcome is the correct detection of the underlying cause (vascular abnormality/lesion) of ICH, sensitivity/specificity analysis and rater reliability, if available.

### Inclusion and exclusion criteria

#### Inclusion criteria

1. Patients presenting with ICH, symptoms of ICH (AVM, dAVF, aneurysm, parenchymal bleed, primary ICH)
2. Participants > 18 years of age
3. Studies with > 15 patients
4. Search date between January 1998 to March 2019
5. Randomised controlled trials (RCT), non-RCT, observational studies
6. Studies with more than one board-certified radiological reviewer
7. Sensitivity and specificity analysis, positive and negative predictive values, ROC (inter-rater agreement)

#### Exclusion criteria

1. Patients presenting with vascular tumours, SAH (sole, with no evidence of parenchymal haemorrhage), trauma, ischemic stroke, post-operative neurosurgery
2. Participants < 18 years of age
3. Studies with < 15 patients
4. Manuscript replies, case studies, guidelines, animal/in vitro studies
5. Studies with only one board-certified radiology reviewer
6. Non-English language studies

### Study selection

The PRISMA guidelines were followed for this study. PubMed, SCOPUS and Web of Science databases were used to search for published studies. The search strategy consisted of Medical Subject Headings (MeSH) terms and text words for primary intracerebral haemorrhage, 4D-CTA, DSA, cerebral haemorrhage, angiography, digital subtraction, arteriovenous malformations, 4D, CTA, dynamic-CTA, time-resolved CTA and CTA.

## Results (Fig. 1)

### Study characteristics

We identified a total of 237 articles. One hundred and twenty-three studies were identified via PubMed using the following MeSH terms: primary intracerebral haemorrhage, spontaneous intracerebral haemorrhage 4D-CTA, DSA, cerebral haemorrhage, angiography, digital subtraction, arteriovenous malformations, 4D, CTA, dynamic-CTA, time-resolved CTA and CTA. Fifty-nine articles were retrieved from Scopus, with 55 articles retrieved from Web of Science using identical search terms as the PubMed review. Duplicate publications (*n* = 126) and articles that failed to meet our inclusion criteria after title and abstract screening were eliminated (*n* = 85). The 26 studies potentially eligible for further analysis were published between 2004 and 2019. Data collection was mainly retrospective (*n* = 20), 4 studies were prospective in nature with a further 2 studies being a clinical audit and a case study respectively. The majority of studies were single centre (*n* = 25), with 1 further study being multi-centre. The 26 articles were potentially viable for analysis based on title and abstract, and all underwent full review of the text. Of the 26 studies identified, 22 articles were excluded on the basis of the studies not performing DSA (*n* = 8) or 4D-CTA (*n* = 7), with a further 5 studies failing to use 4D-CTA and DSA as part of the patient investigations. One study was a service audit, with another study presenting only case studies. The combination of imaging modalities were as follows: standard CTA and DSA (*n* = 4), CTA alone (*n* = 6), 4D-CTA and DSA (*n* = 5), 4D-CTA alone (*n* = 4), CTA, MRA and DSA (*n* = 4), combined 3D-rotational angiography (3D-RA) and DSA (*n* = 1), 3D-CTA and DSA (*n* = 1), 4D-DSA and 2D-DSA (*n* = 1). Sixteen of the 26 studies used DSA as their gold standard (62%), with 10 studies (38%) opting to use imaging signs or clinical outcome as their “gold standard”.

**Fig. 1:**
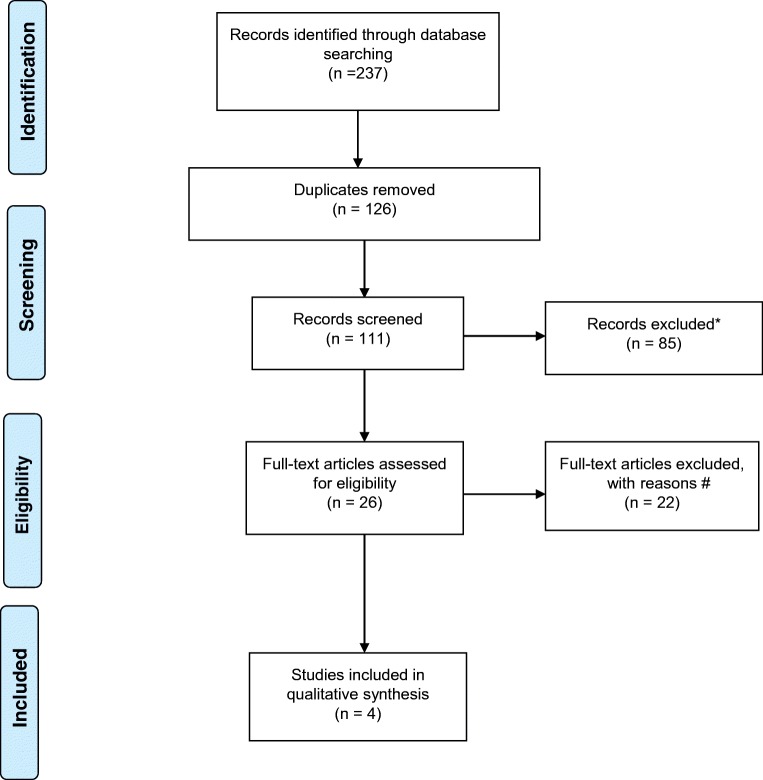
Flowchart of the literature search and study selection of ICH patients, following the Preferred Reporting Items for Systematic Reviews and Meta-Analyses (PRISMA) guidelines (*SAH*, subarachnoid haemorrhage; *TBI*, traumatic brain injury). *Records excluded (*n* = 85) (SAH (*n* = 27), TBI (*n* = 2), case studies (*n* = 10), ischemic stroke (*n* = 6), no 4D-CTA (*n* = 14), no DSA (*n* = 14), review papers (*n* = 7), manuscript response letters (*n* = 1), non-neurological condition (*n* = 2), non-English (*n* = 2)). ^#^Records excluded (*n* = 22): not performing DSA (*n* = 8) or 4D-CTA (*n* = 7), with further 5 studies failing to use 4D-CTA and DSA as part of the patient investigations. One study was a service audit, with another study presenting only case studies

Following the evaluation of the papers, there were only 4 potentially eligible manuscripts; Biswas et al. [5], Wang et al. [12], Willems et al. [15] and Singh et al. [17] for the meta-analysis, with 91 patients (all studies combined) available for analysis (Biswas et al. *n* = 33 patients, Wang and Willems et al. *n* = 17 each, with Singh et al. *n* = 24 patients). Demographics are presented in Table 1. Due to there being limited variability in the sensitivity and specificity of 4D-CTA and DSA in detecting vascular abnormalities (values reached 100% for 3 out of 4 studies), with positive and negative predictive values also reaching 100%, it was not statistically meaningful to perform a meta-analysis. Therefore, the data is presented using only summary statistics and discussed qualitatively.

**Table 1:** Demographics of the 4 studies

Demographics	Author			
	Biswas et al.	Wang et al.	Willems et al.	Singh et al.
Number of patients	33	17	17	24
Mean age (years)	Not specified	37	38 (± 17)	24.8 (± 9.7)
Age range (years)	22–80	20–59	Not specified	23–35
Gender	Not specified	M: 13 F: 4	M: 6 F: 11	M: 15 F: 9
Modality	4D-CTA and DSA	4D-CTA and DSA	4D-CTA and DSA	4D-CTA and DSA
Number of reviewers	2 (CTA) 2 (DSA), 4 in total	2	3	3
Time between CT and DSA (days)	10 (range 0–50 days)	Within 9 days, mean of 1.5 days	4–11 h	Not specified

### Patient demographics

#### Demographics of the patients meeting the reviews search criteria

The age range of all individuals across the 4 studies was 20–80 years. Variability occurred in the time between the initial 4D-CTA examination and the DSA, with the Willems et al. study [15] having a window of 4–11 h, and the Biswas et al. study [5] having a range of 0–50 days between investigations. Table 2 below presents the protocol and radiation dose received by the patient.

**Table 2:** CT and DSA acquisition parameters and radiation doses (frames per second (fps))

Protocol	Author			
	Biswas et al.	Wang et al.	Willems et al.	Singh et al.
4D-CTA				
System	Aquilion One	Aquilion One	Aquilion One	Cine Dynamic Multi 4D
Coverage	16 cm	16 cm	16 cm	20 cm
Detector rows	320 × 0.5 mm	320 × 0.5 mm	320 × 0.5 mm	128 × 0.6 mm
Acquisition	Time density curve (TDC) from test injection	Not specified	Not specified	Not specified
Radiation dose	Mean dose, 6.6 mSv (range, 4–8.3 mSv)	≤ 5.3 mSv	≤ 5.3 mSv	Not specified
DSA				
System	Inova, GE healthcare	Infinix, Toshiba Medical Systems, and ADVANTX LC/LP (GE Medical Systems)	Infinix, Toshiba Medical Systems, and ADVANTX LC/LP (GE Medical Systems)	Allura XPer FB 20/10, Philips Medical Systems
Rate	2.5 fps	3 fps	3 fps	6 fps
Radiation dose	Mean dose, 4.2 mSv (2–10 mSv)	Not specified	Dose varied between 7.89 & 9.12 mSv	Not specified

### Acquisition parameters

#### Imaging parameters and radiation dose for the 4D-CTA and DSA acquisitions

The radiation dose for the 4D-CTA was similar across the Biswas, Wang and Willems studies [5, 12, 15]; the study by Singh et al. [17] did not present the dose acquired using their protocol. Three out of the four studies acquired their 4D-CTA data using an Aquilion One system (Canon Medical Systems Europe). Two studies presented the mean dose during the DSA procedure, which were comparable. 4D-CTA dose was ≤ 5.3 mSv in the Wang et al. study [12]. For the Willems et al. study [15], the dose was recorded as 5.3 mSv or less for the 4D-CTA. The DSA dose in this study was calculated from 4 typical DSA’s, with DSA dose varying between 7.89 and 9.12 mSv. However, it should be noted that the average radiation doses the patient receives can be complex to record for both 4D-CTA and DSA modalities and a comparison of dose between 4D-CTA and DSA is particularly difficult. The variability in the radiation dose received during DSA is attributed to the variable fluoroscopy time per vessel studied, the number of vessels studied, vascular complexity, fluoroscopic parameters and differences in protocols and equipment type [15]. The sensitivity, specificity, positive predictive values (PPV) and negative predictive values (NPV) acquired in the 4 studies comparing the diagnostic ability of 4D-CTA with DSA in the detection of AVM is presented in Table 3 below.

**Table 3:** Sensitivity, specificity, positive predictive values (PPV) and negative predictive value (NPV) for the 4 studies. *McNemar’s test found that there was no statistically significant difference in the detection of AVM or DAVF or both by 4D-CTA and DSA (*p* = 0.25)

	Author					
	Biswas et al.			Wang et al.	Willems et al.	Singh et al.
Number of patients	*33 (total number)	30	3	17	17	24
Modality comparison	4D-CTA and DSA	4D-CTA and DSA	4D-CTA and DSA	4D-CTA and DSA	4D-CTA and DSA	4D-CTA and DSA
Condition	AVM and dural AVF combined	AVM alone	DAVF alone	AVM	AVM	AVM
Sensitivity	77% (95% CI 46–95)	70% (95% CI 35–93%)	100% (95% CI 29–100%)	17/17 (100%)	17/17 (100%)	100%
Specificity	100% (95% CI 83–100)	100% (95% CI 85–100%)	100% (95% CI 88–100%)	17/17 (100%)	17/17 (100%)	100%
PPV	100% (95% CI 69–100)	100% (95% CI 69–100)	100% (95% CI 29–100%)	Not specified	Not specified	100%
NPP	87% (95% CI 66–97)	87% (95% CI 66–97)	100% (95% CI 88–100%)	Not specified	Not specified	100%
Efficacy	91%	91%	100%	Not specified	Not specified	Not specified

#### Sensitivity, specificity, positive predictive values and negative predictive values of 4D-CTA compared with DSA for the 4 studies

Across the four studies, a total of 91 patients were available for analysis. Considering the sensitivity and specificity of 4D-CTA compared with DSA in diagnosing AVM and dAVF, Biswas et al. [5] performed a retrospective review of a group of 33 patients. Out of the 33 patients who underwent both tests, 13 patients were diagnosed as having AVM/DAVF by DSA (10 and 3 respectively). 4D-CTA was able to detect 7 AVM and all 3 DAVF’s. High efficacy (91–100%) was shown between the 4D-CTA and the DSA examinations, which demonstrated that 4D-CTA (using 320-detector row CT) is both practical and has comparable diagnostic accuracy to DSA for diagnosing AVM or DAVF.

However, variability was noted across the studies in the sensitivity of 4D-CTA in the detection of AVM. One hundred percent sensitivity was achieved in the Singh, Wang and Willems studies [12, 15, 17], whereas the Biswas et al. study [5] had a sensitivity of 77% for AVM and dural AVF combined. In this study, the examination of AVM alone resulted in a sensitivity of 70%, and 100% when evaluating DAVF alone. Specificity was 100% for all studies. Positive and negative predictive values were only available for the Biswas and Singh studies [5, 17]. PPV was 100% for both studies, and NPV was 87% in the Biswas et al. study [5] and 100% in the Singh study [17]. Detection of AVM and DAVF alone in the Biswas et al. study [5] yielded an NPV of 88% and 100% respectively.

#### Summary of the capability of 4D-CTA and DSA in diagnosing AVM and determining lesion size (Table 4)

The Wang, Willems and Singh studies [12, 15, 17] demonstrated that 4D-CTA had 100% agreement with DSA in diagnosing an AVM, with all studies reporting that 4D-CTA and DSA were equally as accurate in detecting lesion sizes of < 3 and 3–6 cm. In the Biswas et al. study [5], both 4D-CTA and DSA detected lesion sizes of 3–6 cm, in the cases where the size was < 3 cm, 5 4D-CTA and 8 DSA investigations correctly graded the lesion.

**Table 4:** Detection of lesion size by 4D-CTA and DSA

Author		Biswas et al.		Wang et al.		Willems et al.		Singh et al.	
Modality		4D-CTA	DSA	4D-CTA	DSA	4D-CTA	DSA	4D-CTA	DSA
AVM/DAVF detected		7	10	17	17	17	17	18	18
Lesion size	< 3	5	8	7	7	12	12	13	13
	3–6 cm	2	2	9	9	5	5	5	5
	> 6 cm	0	0	1	1	0	0	N/A	N/A

Wang et al. [12] demonstrated that in a small patient cohort, 4D-CTA had the ability to detect all lesions, including location, size, feeding and draining arteries. Readings were performed by 2 independent raters. In these cases, 4D-CTA demonstrated an equal ability to DSA for distinguishing the main feeding arteries in all patients, achieving a sensitivity and specificity of 100%.

Similarly, the Willems et al. [15] study demonstrated 100% sensitivity and specificity in their patient group (*n* = 17) when comparing 4D-CTA with DSA in patients with an untreated AVM. Three readers evaluated the image data. 4D-CTA detected all AVM, however, difficulties with angioarchitectural details were encountered and were either misinterpreted or missed by 4D-CTA, compared with DSA. However, 4D-CTA was sufficiently accurate for diagnosing and classifying the shunt, although the authors concluded that treatment planning is likely to benefit from accompanying cross-sectional imaging in a variety of planes in combination with perfusion maps [15].

Singh et al. [17] evaluated the accuracy of time-resolved (4D) CTA in comparison with DSA in defining both the morphological and haemodynamic characteristics of AVM required for patient management. Twenty-one patients with a clinical suspicion of AVM and 3 post-AVM diagnosis patients were evaluated in the study. Eighteen patients had a correct diagnosis of AVM, with 3 diagnosed with an AV fistula. Following the comparison of the techniques, the sensitivity and specificity were 100% respectively, with PPV and NPV also being 100% for both procedures. The Singh study [17] reported that early venous filling was present in all 21 cases, independent of nidus size. An accurate distinction between feeding artery territories was made in cases where the shunt was detected using 4D-CTA. The authors further noted that 4D-CTA was fully concordant in depicting flow characteristics, venous drainage, and establishment of the site and grade of venous stenosis. However, limitations were again noted when examining finer architectural details. Issues highlighted in this study included impaired spatial and temporal resolution and reduced SNR when compared with DSA. As noted in the other three comparative studies, 4D-CTA is accurate when classifying and identifying major angioarchitecture features (e.g. arterial feeders, aneurysms, nidus characteristics and venous drainage), but can encounter issues when evaluating finer angioarchitecture and haemodynamics, which has the potential to be misinterpreted or missed [17].

## Discussion

In this study, we conducted an extensive literature review to determine the number of studies available comparing the accuracy of 4D-CTA with DSA in the detection of potential underlying vascular causes of spontaneous ICH, for example, the presence of an AVM. Haemorrhagic stroke resulting from the rupture of an AVM is a serious clinical complication and is the leading cause of intracerebral haemorrhage in young adults, which can result in functional disabilities or even death in these patients [12]. AVM and other vascular malformations are complex, with several treatment options available to patients, ranging from conservative clinical management, stereotactic radiosurgery, open neurosurgery, endovascular embolization or a combination of options. It is essential that the AVM is characterised and diagnosed as quickly as possible to ensure optimal treatment. Invasive cerebral DSA allows the accurate analysis of the nidus angioarchitecture enabling the identification and distinction of feeding arteries and draining veins present in AVMs, due to the high temporal and spatial resolution present with this technique. However, despite the high resolution, this technique is invasive in nature, requires extensive resources and is associated with a small risk of major complication (≤ 1.3%), which includes mortality (≤ 0.1%) [17]. At the present time, DSA remains the gold standard for the evaluation of cerebral vasculature due to its increased resolution.

To our knowledge, there are no studies available that have performed a meta-analysis comparing 3D or 4D-CTA with DSA in the detection of underlying causes of ICH. However, following the systematic review, it was concluded that there were few suitable papers available for meta-analysis, with only 4 studies identified from the review, which aimed to assess the ability of 4D-CTA in providing comparative morphological and temporal information of the AVM compared to DSA, to determine whether 4D-CTA has the potential to replace catheter angiography when diagnosing or following-up cerebral AVM’s. We were unable to include 3D-CTA into the analysis, due to this technique providing limited information relating to flow dynamics, which is therefore not equivalent to 4D-CTA.

Meta-analyses performed before the technical developments of 3D-CTA and 4D-CTA directly compared standard CTA with DSA in the detection of cerebral aneurysms [18]. Results from this analysis concluded that DSA remained the gold-standard technique. However, other reviews comparing CTA with DSA have reported that CTA was as good if not better than DSA in diagnosing, excluding and treating cerebral aneurysms [5, 9], having the added benefits of a reduced risk of adverse events, improved patient comfort, wider availability and cost-effectiveness.

More recent studies have demonstrated an improvement in the diagnostic accuracy of CTA (3D-CTA and 4D-CTA) techniques for detecting underlying vascular abnormalities [5, 9, 15, 19]. The improvement in 4D-CTA combines the traditional 3D-CTA with the 4th dimension of time to create a stereoscopic image continuously displaying blood vessels within the short interval of time taken for one investigation. Compared with traditional CTA, which cannot identify AVM vessels during different blood flow phases, 4D-CTA is capable of displaying vascular lesions in the arterial, capillary and venous phases of blood flow through its time-resolved techniques and is therefore a reflection of the vessel’s characteristics at each phase [12, 15]. 4D-CTA has the potential to exclude the vascular causes of haematoma in many cases, therefore avoiding unnecessary DSA investigations. Other major advantages of using 4D-CTA as the initial investigation include the availability of this technique, with many district general hospitals (DGH) involved in hyperacute stroke care having modern CT scanners (minimum of 256 detector rows) that potentially have the capability to run this examination. This avoids unnecessary patient transfer to specialist centres for tests unless deemed to be clinically necessary, such as cases where 4D-CTA indicates that a DSA will be required as part of the patient’s treatment plan. The wider implementation of 4D-CTA includes faster diagnosis due to wider availability and a relatively short scan time, cost-effectiveness, improved anatomical visualisation (increased sensitivity) for more rapid treatment planning, with reduced morbidity and mortality compared with DSA. The disadvantages of CTA are largely historical, including previous reductions in sensitivity in comparison with DSA, reduced availability in DGH, with older techniques providing incomplete information relating to flow patterns in some vascular areas, such as the Circle of Willis [20]. These previous disadvantages have been addressed and overcome by modern CTA techniques.

Discrepancies noted between 4D-CTA and DSA, as identified in the Biswas et al. study [5] appeared to be interpretation errors when primarily reported, as the lesions were detected following a review of thin section images. Two lesions were visible on the MIP image, although they had poor visibility and were more conspicuous on thinner section images. The 100% specificity in the detection of AVM or DAVF by 4D-CTA indicates that it could be an effective tool in excluding vascular conditions and potentially obviating DSA in the future. 4D-CTA provides a large data set of images and thorough review of all images may help to reduce false-negative cases. For future studies, it would be prudent to assess inter-rater reliability to avoid the introduction of any potential errors that may be producing the observed variability [19]. The level of training for reading 4D-CTA scans should also be standardised across centres to reduce any potential discrepancies, particularly in non-specialist district general hospitals. Additionally, the small number of patients included in the studies reviewed renders it difficult to reach a definite conclusion, which warrants further large-scale studies in this area.

## Conclusion

Due to the restricted number of patients and study heterogeneity for 3 out of the 4 studies (100% sensitivity and specificity were reached), performing a meta-analysis was not feasible. Therefore, the literature was assessed qualitatively. The outcome from the 4 studies evaluated above has demonstrated that in the majority of patient cases, 4D-CTA is equally as good as IA-DSA in excluding the vascular causes of haematoma, although there are still issues with the classification of the finer angioarchitectural structures. The lack of relevant studies and the small number of patients included in the four studies reviewed has also highlighted that it would be advantageous to perform further studies comparing 4D-CTA with DSA in larger prospective patient cohorts to increase the evidence base which would inform clinical decision making in patients with spontaneous ICH.

## Abbreviations

4D-CTA: Four-dimensional CTA
DSA: Digital subtraction angiography
ICH: Intracerebral haemorrhage
AVM: Arteriovenous malformation
DAVF’s: Dural arteriovenous fistulae
MCA: Middle cerebral artery
mSv: MilliSieverts
PPV: Positive predictive value
NPV: Negative predictive value
